# Determinants, inequalities, and spatial patterns of diarrhea in the Peruvian under-five population: findings from nationally representative survey data

**DOI:** 10.3389/fpubh.2023.1170670

**Published:** 2023-06-27

**Authors:** Akram Hernández-Vásquez, Rodrigo Vargas-Fernández, Efrain Y. Turpo Cayo

**Affiliations:** ^1^Centro de Excelencia en Investigaciones Económicas y Sociales en Salud, Vicerrectorado de Investigación, Universidad San Ignacio de Loyola, Lima, Peru; ^2^Universidad Científica del Sur, Lima, Peru; ^3^Universidad Nacional Agraria La Molina, Lima, Peru

**Keywords:** diarrhea, child, cross-sectional studies, health inequities, spatial analysis, Peru

## Abstract

**Objective:**

To determine the associated factors, decompose the socioeconomic inequalities, and analyze the spatial distribution of childhood diarrhea in Peru.

**Methods:**

A cross-sectional analytical study was conducted using data from the National Demographic and Family Health Survey 2021. The dependent variable was the presence of diarrhea in the last two weeks. Three types of analysis were performed: (i) to evaluate the associated factors, generalized linear models of the Poisson family with logarithmic link were applied and prevalence ratios with their 95% confidence intervals were reported; (ii) for the analysis of inequalities, a decomposition of the Erreygers concentration index was performed using a generalized linear model; and (ii) a spatial autocorrelation analysis, hot spot analysis and cluster and outlier analysis were performed.

**Results:**

A total of 18,871 children under 5 years of age were included. The prevalence of diarrhea in this population was 10.0%. Determinants such as being aged 0–23 months, being male, belonging to the poorest, poorer and rich wealth index, and residing in the Highlands and Jungle increased the probability of presenting diarrhea. In the decomposition analysis, diarrhea had a pro-poor orientation, with the greatest contributors were age 0–23 months, belonging to the poorest and poorer wealth indexes, and residing in the Highlands and Jungle. Spatial analysis showed that the highest concentrations and occurrence of this event were observed in departments of the Highlands and Jungle.

**Conclusion:**

Government institutions seeking to reduce the numbers and burden of childhood diarrhea should focus their strategies on promoting hygiene measures and improving access to water and sanitation services, especially in poor populations living in the Peruvian Highlands and Jungle.

## 1. Introduction

Childhood diarrhea is a global health problem, which has negative consequences such as stunted growth, impaired cognitive development, and increased risk of mortality in children under 5 years of age (1, 2). Despite the World Health Organization (WHO) and the United Nations Children’s Fund seeking to reduce child deaths from diarrhea by 2025 through a global action plan (3), more than 1 billion children suffer from an episode of diarrhea each year, more than 400,000 children died from this cause, and more than 40 million disability-adjusted life years were generated in 2016 (4). These figures expose an economic cost for health care systems due to the high proportion of patients requiring therapeutic measures during outpatient care and hospitalization, and economic cost to households due to the acute and chronic consequences diarrhea produces in children (5). In this sense, the biomedical literature has described cost-effective measures for the prevention of diarrhea, such as oral rehydration therapy, improvements in water supply and sanitation systems, hand hygiene, vaccination against rotavirus (the most common pathogen in children), and dietary supplementation, and these measures would be useful, especially in countries with limited economic resources (6–9).

Although childhood diarrhea is a preventable and treatable disease (10), low and middle-income countries (LMIC) have the highest mortality rates due to childhood diarrhea, with 90% of deaths from this cause occurring in sub-Saharan Africa (185.7 deaths per 100,000 population) and South Asia (66.4 deaths per 100,000 population) (4). These figures may reflect the burden of factors that promote the occurrence of childhood diarrhea in LMICs, such as poor access to health services, lack of access to safe water and sanitation, and living in poverty (10). In Latin America and the Caribbean, more than 8,000 deaths and more than 140 million episodes of diarrhea occurred in 2016 (4). Specifically, in 2017, the highest mortality figures were observed in countries such as Haiti (3,140 deaths), Brazil (1,800 deaths), Guatemala (1,290 deaths), and Mexico (1,250 deaths), with Guatemala presenting one of the highest incidences that same year (11). However, evidence indicates that there are spatial and temporal variations within countries that generate differences in prevalence, incidence and mortality figures for childhood diarrhea (11).

In Peru, differences by geographic position have been reported in the mortality rate for childhood diarrhea, with 34 provinces (mainly in the Highlands and Jungle regions) having mortality rates around 20% higher than the country average (four deaths per 100,000 children) in 2017 (11). On the other hand, the biomedical literature has described socioeconomic, environmental and behavioral factors that increase the probability of suffering an episode of childhood diarrhea in developing countries (12, 13). These factors are related to male sex and older age of the child, number of children in the household, residing in a rural area, inadequate waste disposal practices, lack of handwashing facilities, lack of drinking water sources, lack of vaccination against rotavirus, and inadequate breastfeeding practices (12, 13). Likewise, another factor related to the occurrence of childhood diarrhea is living in conditions of poverty, with wealthier families having greater opportunities for hand hygiene with soap and greater availability of latrines and adequate water supplies in the home (14–16). In this sense, the WHO seeks to promote policies and investments for increased access to quality drinking water, sanitation and hygiene in the most vulnerable populations (17).

Although the report of the National Demographic and Family Health Survey (ENDES - acronym in Spanish) reported that the prevalence of childhood diarrhea in 2021 has decreased by about six percentage points since 2010 (18), this disease continues to be one of the main causes of morbidity and mortality in the Peruvian child population (19). However, there is little scientific evidence on the associated factors, socioeconomic inequalities, or the geographic distribution of diarrhea in Peru, with only reports of morbidity and mortality figures in periods prior to the present study. Therefore, the objective of the present study was to determine the associated factors, decompose the socioeconomic inequalities and analyze the spatial distribution of childhood diarrhea in Peru.

## 2. Materials and methods

### 2.1. Study design, data source, and sampling

A cross-sectional analytical study was conducted using data from the ENDES 2021 (18). The ENDES is part of the national surveys developed by the Measure Demographic and Health Surveys (DHS) in several LMIC. In general, the ENDES 2021 is a national survey conducted annually by the National Institute of Statistics and Informatics of Peru (INEI - acronym in Spanish) that collects information on the household, women of childbearing age, and children. It also includes a health questionnaire aimed at people aged 15 years and older and children under 12 years of age (18).

The ENDES 2021 sampling was probabilistic, two-stage, stratified and independent at both the departmental level and area of residence (urban and rural) (18, 20). In the urban area, the sampling units consisted of clusters and private dwellings. In the rural area, the sampling units were the rural census area and private dwellings. The research unit of the survey was the usual residents of private dwellings who spent the night before the survey in the selected dwelling (20). More methodological information about the ENDES 2021 can be found in the technical report and the final report of the survey (18, 20).

### 2.2. Inclusion/exclusion criteria

**Figure 1:**
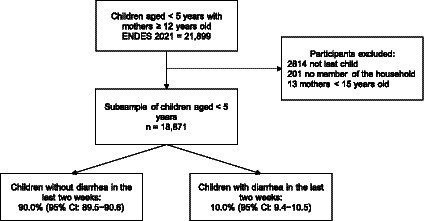
Flow chart of participants included in the study.

The present study included children under 5 years of age whose mothers responded to the question on the occurrence of diarrhea during the two weeks prior to the survey. In the case of mothers with two or more children under 5 years of age, the last child was included in the study. Finally, children no member of the household and with mothers <15 years old were excluded, and a total of 18,871 children under 5 years of age were included (Figure 1).

### 2.3. Variables

The outcome variable was the history of diarrhea in the last two weeks. This variable was coded as 1 if the mother reported that her child had diarrhea in the last two weeks prior to the survey and 0 if she did not.

The individual variables included in the analysis of association and inequality were the following: age group of the child in months (36–59, 24–35, and 0–23), sex of the child (female, male), age group of the mother in years (15–24, 25–34, and 35–49), educational level of the mother (up to primary, secondary, and higher), area of residence (urban, rural), region of residence (Coast, Highlands, and Jungle), type of sanitation (not improved, improved), and supply of drinking water (unimproved, improved). The construction of the type of sanitation and supply of drinking water variables was based on the statistical guide of the DHS program (21). Improved sanitation included sewerage system, septic tank, ventilated latrine, and pit latrine, while unimproved sanitation included open latrine, river/canal, unserved, and others. On the other hand, improved drinking water supply consisted of indoor and outdoor supply, well in the house or yard, public tap, tanker truck, bottled water and rainwater, while unimproved supply consisted of river/drought/lagoon, spring and others. The characterization of socioeconomic level was based on the household wealth index. This variable is calculated in the ENDES database and follows the methodology established by the DHS Program, which is based on the characteristics of housing and household assets (22). Further methodological details about the construction of the wealth index can be found in the report elaborated by Rutstein et al. (22). This index was included in the study as a categorical variable divided into quintiles (poorest, poorer, middle, richer, and richer) and as a continuous variable for the analysis of associated factors and inequalities, respectively.

The variables used for the spatial analysis were the latitude and longitude of the cluster where the home of the child under 5 years of age was located. For each of these points, the number of children with and without diarrhea was counted. In the ENDES 2021, the geolocation measurement (latitude and longitude) is given by taking points in the global positioning system (GPS) measured with a tablet 1 m from the front door of the informant’s dwelling (23). More information about the measurement of the geolocation of dwellings in the ENDES 2021 can be found in the survey manual (23).

### 2.4. Statistical analysis

Descriptive, associated factors and inequalities analyses were performed in Stata 17 (StataCorp, College Station, TX, United States). Analyses were sample-weighted and the complex stratified, two-stage, clustered sampling design of ENDES 2021 was considered. Significance was set for a two-sided *p* value at <0.05.

In the descriptive analysis, the weighted frequencies of the study variables were reported and the chi-square test with Rao-Scott correction was applied to determine differences between the proportions of the independent and dependent variables. To evaluate the associated factors, generalized linear models of the Poisson family with logarithmic link (crude and adjusted) were applied to evaluate the association between the independent variables and the occurrence of diarrhea in the last two weeks in children under 5 years of age and to report crude (PR) and adjusted (aPR) prevalence ratios with their 95% confidence intervals (95% CI). Variables included in the adjusted analysis were selected when they obtained a *p* value <0.20 in the crude analysis. Multicollinearity among the independent variables was checked using the *collin* command in Stata, and no values were obtained in the variance inflation factor indicating multicollinearity.

The analysis of inequalities is presented by means of a decomposition of the Erreygers concentration index calculated by means of the *conindex* command in Stata (24). For this purpose, this index was decomposed by means of a generalized linear model in order to establish the percentage contribution of the independent variables to the inequality of the occurrence of the event studied. Likewise, the elasticity, concentration, and contribution to inequality are reported for each independent variable (25). Elasticity denotes the change in the outcome of interest associated with a one-unit change in the independent variable. The concentration index represents the concentration index of the independent variables with reference to the wealth index. The contribution and percentage contribution represent the absolute and relative contribution of each independent variable included in the model to the overall socioeconomic inequality in the outcome of interest. A positive or negative value in the contribution or percentage contribution results in an increase or decrease in inequality (25).

The geographic information system program ArcGIS Desktop version 10.5 (ESRI Inc., Redlands, CA, United States) was used to perform the spatial autocorrelation analysis (Moran’s Global Index), hot spot analysis (Getis-Ord Gi*), and cluster and outlier analysis (Anselin Local Moran’s I) using 3,244 clusters. For the district analysis, the sum of the clusters per district was performed, obtaining 1,342 district data. In our study, the Moran’s Global Index assessed the overall pattern and trend of diarrhea cases to determine whether they were clustered, scattered, or random. The Moran’s Global Index is bounded between −1 and + 1, where a positive value indicates a spatially clustered pattern, 0 indicates a randomly distributed spatial pattern, and a negative value indicates a dispersed pattern. An interpolation analysis was also performed, choosing the Inverse Distance Weighting model in view of the non-normality of the data distribution to apply an ordinary Kriging analysis. Finally, using the SaTScan V10.1 program (Martin Kulldorff, Boston, MA, United States) (26), a purely spatial Kulldorff scanning analysis (Bernoulli model) was applied to identify clusters with high risk of diarrhea occurrence in the last two weeks (27). This method employs the creation of a circular window that scans the study area, where the radius of the circle, depends on the maximum size of the population considered for the clusters. In this study, a maximum of 25% was set, i.e., a cluster can contain at most 25% of the total population with diarrhea cases (27). The results are reported as log likelihood ratio (LLR) and value of *p*, in which it is interpreted that the risk inside the window is different from that outside the window. The value of *p* is calculated through the Monte Carlo simulation model with 999 replicates. A low value of *p* suggests that the identified cluster is significant and probably not the result of chance. If the LLR value is positive and high it indicates a possible spatial concentration of cases (27).

### 2.5. Ethical considerations

The ENDES 2021 databases are freely available on the INEI website: https://iinei.inei.gob.pe/microdatos/. Since these are anonymized data that are in the public domain, approval by an ethics committee was not requested.

## 3. Results

### 3.1. Characteristics of the population included

Data from 18,871 children under 5 years of age were analyzed. The largest proportion of children ranged in age from 0 to 23 months (43.4%) and 9,563 (50.8%) were female. It should be noted that most of the mothers of these children were between 25 and 34 years of age (47.5%) and had a secondary education (46.0%). Further details of the characteristics of the population are shown in Table 1.

**Table 1:** Characteristics of the children under 5 years of age included in the study (*n* = 18,871).

Characteristics	*n*	%
Age groups of child (months)		
36–59	6,907	36.7
24–35	3,842	19.9
0–23	8,122	43.4
Child sex		
Female	9,308	49.2
Male	9,563	50.8
Age group of mother (years)		
15–24	2,672	20.6
25–34	5,136	47.5
35–49	2,118	31.9
Educational level of mother		
Higher	6,439	37.1
Secondary	8,965	46.0
Up to primary	3,467	16.9
Wealth index		
Richest	1,869	14.8
Richer	2,811	18.0
Middle	3,735	21.3
Poorer	5,017	23.0
Poorest	5,439	22.9
Area of residence		
Urban	13,090	76.4
Rural	5,781	23.6
Region of residence		
Coast	7,970	56.5
Highlands	6,237	26.6
Jungle	4,664	17.0
Type of sanitation		
Unimproved	4,999	22.1
Improved	13,872	77.9
Supply of drinking water		
Unimproved	1,240	5.6
Improved	17,631	94.4

Estimates include the weights and ENDES 2021 sample specifications.

Rounding cannot add up to 100.

### 3.2. Population characteristics according to the prevalence of diarrhea in 2021

The prevalence of diarrhea in children under 5 years of age in the last two weeks was 10.0% in 2021. The highest proportion of children presenting diarrhea was found in the 0- to 23-month age group, and they were male (13.4%). In addition, the highest proportion of mothers were in the 15–24 age group (12.7%) and had secondary education (10.9%). Regarding the households of children presenting diarrhea, the largest proportion belonged to the poorest (12.8%) and poor (11.8%) wealth indexes, resided in a rural area (11.7%), in the Jungle region (14.6%), and had unimproved sanitation (12.3%) and drinking water supply (15.0%; Table 2).

**Table 2:** Prevalence of diarrhea by characteristics of children under five years of age included in the study.

	Diarrhea		
	No (*n* = 16,831)	Yes (*n* = 2,040)	
Characteristics	% (95% CI)	% (95% CI)	*p* value^*^
Overall	90.0 (89.5–90.6)	10.0 (9.4–10.5)	
Age groups of child (months)			
36–59	94.5 (93.8–95.1)	5.5 (4.9–6.2)	<0.001
24–35	89.2 (88.0–90.4)	10.8 (9.6–12.0)	
0–23	86.6 (85.7–87.5)	13.4 (12.5–14.3)	
Child sex			
Female	90.6 (89.8–91.3)	9.4 (8.7–10.2)	0.041
Male	89.5 (88.7–90.2)	10.5 (9.8–11.3)	
Age group of mother (years)			
15–24	87.3 (86.0–88.6)	12.7 (11.4–14.0)	<0.001
25–34	89.7 (88.9–90.5)	10.3 (9.5–11.1)	
35–49	92.3 (91.5–93.0)	7.7 (7.0–8.5)	
Educational level of mother			
Higher	91.3 (90.4–92.1)	8.7 (7.9–9.6)	0.001
Secondary	89.1 (88.3–89.9)	10.9 (10.1–11.7)	
Up to primary	89.7 (88.3–90.9)	10.3 (9.1–11.7)	
Wealth index			
Richest	93.8 (92.4–94.9)	6.2 (5.1–7.6)	<0.001
Richer	90.9 (89.5–92.1)	9.1 (7.9–10.5)	
Middle	91.7 (90.6–92.7)	8.3 (7.3–9.4)	
Poorer	88.2 (87.0–89.3)	11.8 (10.7–13.0)	
Poorest	87.2 (86.0–88.3)	12.8 (11.7–14.0)	
Area of residence			
Urban	90.6 (89.9–91.2)	9.4 (8.8–10.1)	<0.001
Rural	88.3 (87.2–89.4)	11.7 (10.6–12.8)	
Region of residence			
Coast	91.9 (91.1–92.6)	8.1 (7.4–8.9)	<0.001
Highlands	89 (88.0–90.0)	11 (10.0–12.0)	
Jungle	85.4 (84.0–86.7)	14.6 (13.3–16.0)	
Type of sanitation			
Unimproved	87.7 (86.6–88.8)	12.3 (11.2–13.4)	<0.001
Improved	90.7 (90.0–91.3)	9.3 (8.7–10.0)	
Supply of drinking water			
Unimproved	85.0 (82.4–87.4)	15.0 (12.6–17.6)	<0.001
Improved	90.3 (89.8–90.9)	9.7 (9.1–10.2)	

Estimates include the weights and ENDES 2021 sample specifications.

* The value of *p* was calculated using the Rao-Scott Chi-squared test.

CI, confidence interval.

### 3.3. Factors associated with the presence of diarrhea in children under 5 years of age in 2021

In the adjusted analysis, it was found that children aged 0–23 months (aPR: 2.30; 95% CI: 2.01–2.62) and 24–35 months (aPR: 1.91; 95% CI: 1.62–2.24), being male (aPR: 1.12; 95% CI: 1.01–1.25), and households of children belonging to the poorest (aPR: 1.69; 95% CI: 1.27–2.24), poorer (aPR: 1.57; 95% CI: 1.23–2.00), and richer (aPR: 1.34; 95% CI: 1.05–1.72) wealth indexes, and located in the Highlands (aPR: 1.27; 95% CI: 1.09–1.46) and Jungle (aPR: 1.59; 95% CI: 1.38–1.83) increased the probability of presenting diarrhea in children under 5 years of age in the last two weeks. In contrast, children with mothers belonging to the 35–49 age group (aPR: 0.79; 95% CI: 0.68–0.92), and households of children located in a rural area (aPR: 0.83; 95% CI: 0.72–0.95), and had an improved drinking water supply (aPR: 0.82; 95% CI: 0.68–0.98) decreased the probability of presenting diarrhea (Table 3).

**Table 3:** Factors associated with diarrhea among children under 5 years of age in Peru.

Characteristics	Crude model		Adjusted model	
	PR (95% CI)	*p* value	aPR (95% CI)	*p* value
Age groups of child (months)				
36–59	Reference		Reference	
24–35	1.95 (1.66–2.30)	<0.001	1.91 (1.62–2.24)	<0.001
0–23	2.43 (2.13–2.77)	<0.001	2.30 (2.01–2.62)	<0.001
Child sex				
Female	Reference		Reference	
Male	1.12 (1.00–1.24)	0.042	1.12 (1.01–1.25)	0.029
Age group of mother (years)				
15–24	Reference		Reference	
25–34	0.81 (0.72–0.92)	0.001	0.94 (0.83–1.07)	0.362
35–49	0.61 (0.53–0.70)	<0.001	0.79 (0.68–0.92)	0.002
Educational level of mother				
Higher	Reference		Reference	
Secondary	1.25 (1.11–1.41)	<0.001	1.05 (0.92–1.19)	0.482
Up to primary	1.19 (1.02–1.40)	0.032	0.91 (0.76–1.08)	0.279
Wealth index				
Richest	Reference		Reference	
Richer	1.46 (1.14–1.87)	0.003	1.34 (1.05–1.72)	0.018
Middle	1.33 (1.04–1.70)	0.022	1.17 (0.91–1.50)	0.210
Poorer	1.90 (1.52–2.38)	<0.001	1.57 (1.23–2.00)	<0.001
Poorest	2.05 (1.64–2.57)	<0.001	1.69 (1.27–2.24)	<0.001
Area of residence				
Urban	Reference		Reference	
Rural	1.24 (1.10–1.39)	<0.001	0.83 (0.72–0.95)	0.008
Region of residence				
Coast	Reference		Reference	
Highlands	1.35 (1.19–1.54)	<0.001	1.27 (1.09–1.46)	0.001
Jungle	1.80 (1.58–2.05)	<0.001	1.59 (1.38–1.83)	<0.001
Type of sanitation				
Unimproved	Reference		Reference	
Improved	0.76 (0.68–0.85)	<0.001	1.02 (0.89–1.17)	0.789
Supply of drinking water				
Unimproved	Reference		Reference	
Improved	0.65 (0.54–0.77)	<0.001	0.82 (0.68–0.98)	0.032

Estimates include the weights and ENDES 2021 sample specifications.

* Adjusted for the variables shown in column.

PR, prevalence ratio; aPR, adjusted prevalence ratio; CI, confidence interval.

### 3.4. Decomposition analysis of inequality in the distribution of diarrhea in children under 5 years of age in 2021

Table 4 shows that children aged 0–23 months (−0.0327), with mothers that had primary (−0.5321) and secondary (−0.0998) education, household of children belonging to a poorest (−0.7715) and poorer (−0.3125) wealth indexes, located in a rural area (−0.6724), and in the Highlands (−0.3128) and Jungle (−0.3815) regions presented negative concentration indexes, indicating that they were concentrated in the poorest quintiles. Likewise, it was found that the greatest contributors to explaining the inequality gap were being 0–23 months old (9.4%), household of children belonging to the poorest (74.6%) and poorer (25.9%) wealth indexes, and located in the Highlands (15.8%) and Jungle (24.8%) regions.

**Table 4:** Decomposition of concentration indices of diarrhea between children under five in Peru.

Characteristics	Elasticity	Concentration index	Contribution	% Contribution
Age groups of child (months)				
36–59	Reference	Reference	Reference	Reference
24–35	0.0123	0.0078	0.0004	−0.8
0–23	0.0349	−0.0327	−0.0046	9.4
Child sex				
Female	Reference	Reference	Reference	Reference
Male	0.0058	0.0050	0.0001	−0.2
Age groups of mother (years)				
15–24	Reference	Reference	Reference	Reference
25–34	−0.0029	0.0173	−0.0002	0.4
35–49	−0.0075	0.0783	−0.0024	4.9
Educational level of mother				
Higher	Reference	Reference	Reference	Reference
Secondary	0.0021	−0.0998	−0.0009	1.8
Up to primary	−0.0017	−0.5321	0.0036	−7.3
Wealth Index				
Richest	Reference	Reference	Reference	Reference
Richer	0.0051	0.5238	0.0106	−21.8
Middle	0.0031	0.1310	0.0016	−3.4
Poorer	0.0101	−0.3125	−0.0126	25.9
Poorest	0.0117	−0.7715	−0.0362	74.6
Area of residence				
Urban	Reference	Reference	Reference	Reference
Rural	−0.0045	−0.6724	0.0121	−25.0
Region of residence				
Coast	Reference	Reference	Reference	Reference
Highlands	0.0061	−0.3128	−0.0077	15.8
Jungle	0.0079	−0.3815	−0.0121	24.8
Type of sanitation				
Unimproved	Reference	Reference	Reference	Reference
Improved	0.0015	0.1795	0.0011	−2.2
Supply of drinking water				
Unimproved	Reference	Reference	Reference	Reference
Improved	−0.0198	0.0424	−0.0034	6.9
Residual 0.0018				

Estimates include the weights and ENDES 2021 sample specifications.

### 3.5. Spatial analysis of diarrhea in children under 5 years of age in 2021

**Figure 2:**
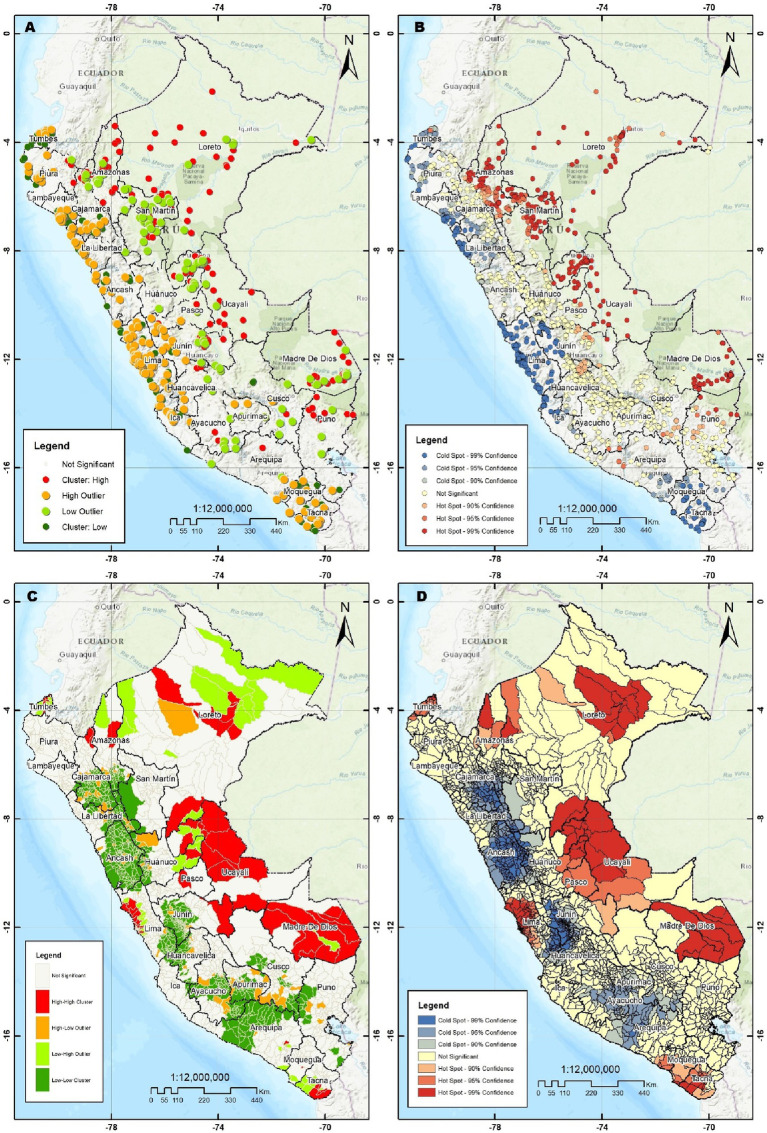
**(A)** Cluster and outlier analysis by Anselin Local Moran Index. **(B)** Hot spot Analysis Getis Ord Index. **(C)** Cluster and outlier analysis across districts by Anselin Local Moran Index. **(D)** Hot spot analysis across districts by Getis Ord Gi.

The spatial autocorrelation of clusters was positive and statistically significant (Moran’s Index 0.0461, *p* < 0.001 and *z*-score 9.34). Likewise, the spatial autocorrelation of clusters at the district level was positive and significant in the year 2021 (Moran’s Index 0.1385, *p* < 0.001 and *z*-score 5.77; see Supplementary material). Figure 2 shows that the highest concentrations of children under 5 who had diarrhea in the last two weeks were found in the departments of Loreto, Amazonas, Ucayali, Madre de Dios, and Pasco, which belong to the Highlands and Jungle regions of Peru. In addition, it is observed that the high priority departments (high-high hotspot) were Loreto, Amazonas, Ucayali, Pasco, Lima, Madre de Dios, Junín, and Tacna.

**Figure 3:**
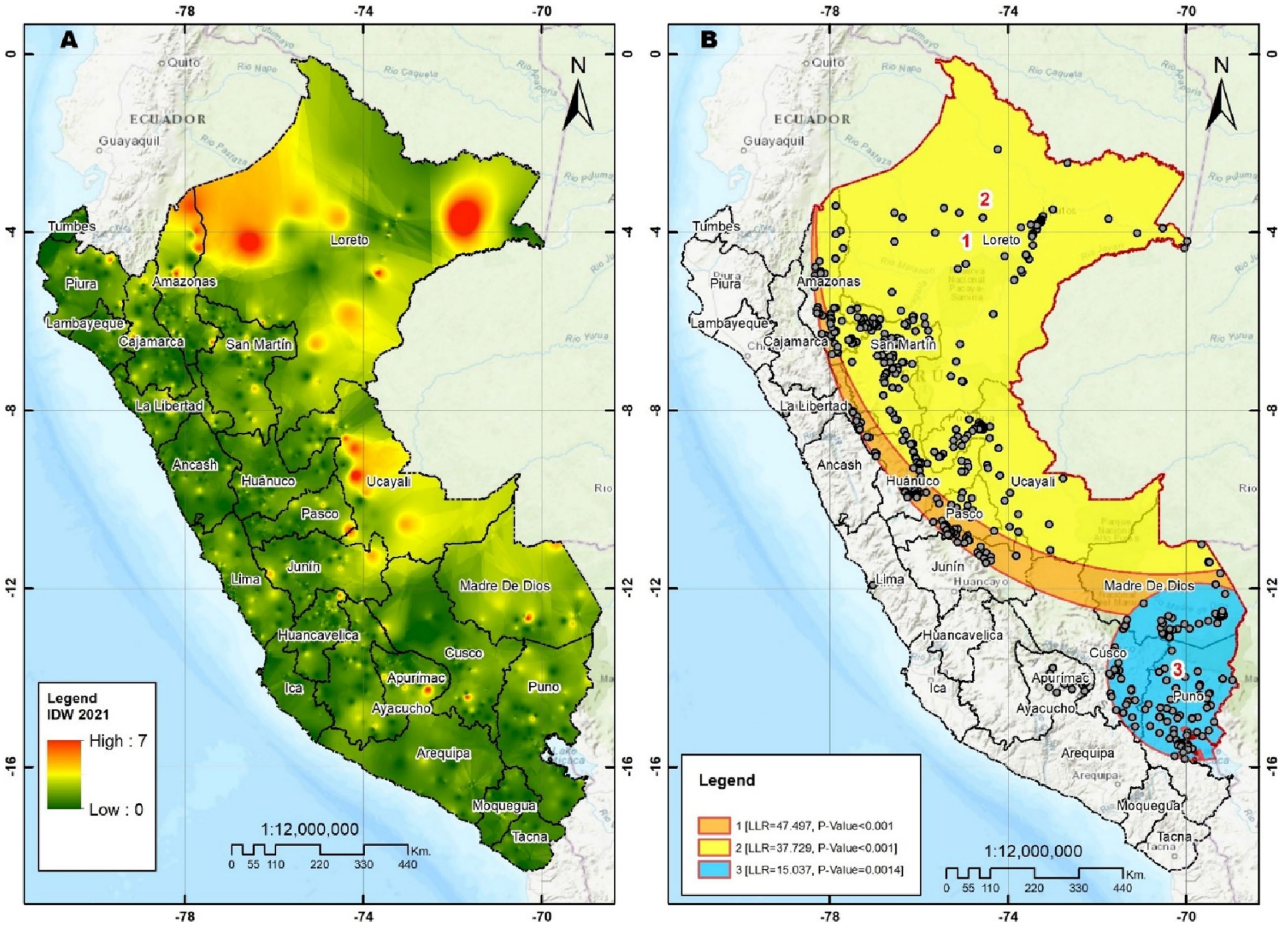
**(A)** Interpolation inverse distance weighting. **(B)** Spatial SaTScan analysis.

Figure 3 shows that the departments with the highest risk of diarrhea in children under 5 years of age were located in regions belonging to the Jungle (Loreto, Amazonas, Ucayali, and Madre de Dios), which is corroborated by an LLR value of 47.497 and 37.729 (*p* < 0.001) in these departments. In addition, the north of the Puno region and proximities with the regions of Cusco and Madre de Dios also had a spatial concentration of diarrhea cases with an LLR value equal to 15.037 (*p* = 0.001).

## 4. Discussion

According to our findings on the prevalence of childhood diarrhea, it is possible to determine that one in 10 Peruvian children had an episode of diarrhea in the two weeks previous to the survey in 2021. In addition, we found that the main determinants that increased the probability of having childhood diarrhea were the age and sex of the child, wealth index, and region of residence, while the determinants that decreased the probability were related to the age of the mother, area of residence, and improved drinking water supply. Inequality decomposition analysis showed that childhood diarrhea had a pro-poor orientation, in which the main contributors were related to child age, wealth index, and region of residence. Finally, spatial analysis identified that the clusters with the highest frequencies and risk of occurrence of childhood diarrhea were concentrated in the Highlands and Jungle regions.

The prevalence of childhood diarrhea in the last two weeks was found to be 10% in 2021. This result is lower than that reported in studies conducted in Ethiopia (22%), Sub-Saharan Africa (15.3%), and Mesoamerica (13%) (28–30) but is higher than that reported in studies conducted in India (9.2%), Bangladesh (5.7%), and Vietnam (7%) (31–33). In Peru, according to the official ENDES report, the prevalence of childhood diarrhea decreased over time from 1986 (29.5%) to 2010 (15.2%) and 2020 (8.2%) (18). Our finding shows an interruption of this decreasing trend that could be attributed to various constraints experienced by the child population with the COVID-19 pandemic (increasing household poverty and slowing the achievement of the Sustainable Development Goals) and previous socioeconomic conditions (34). These constraints are related to the limited capacity of government institutions to provide efficient sanitation and drinking water supply. A study conducted in the Peruvian territory reported that the enterobacterium *Escherichia coli* was found in the water for human consumption in one out of four households, indicating the presence of a pathogen that causes diarrheal diseases in the population (35). In addition, it has been reported that other limitations include inadequate practice of handwashing (due to a greater number of children in the home) (36) and climatic changes (especially an increase in environmental temperature) perceived in Peru (37), thereby producing an increase in the prevalence of childhood diarrhea. In this regard, government institutions should redouble their efforts to ensure compliance with Sustainable Development Goal 6, which seeks to improve access to and quality of water in the population and, consequently, to reduce the burden of disease due to childhood diarrhea and the costs of this disease to the health system (5).

In relation to the associated factors, it was found that some characteristics such as children aged 0–35 months, being male, belonging to the poorest wealth index, and residing in the Highlands and Jungle increased the probability of presenting childhood diarrhea. These findings are similar to those reported in studies conducted in Mesoamerica, Iraq, Vietnam, and India (28, 31, 33, 38), which reported that being 6–23 months old, being male, and belonging to the poorest wealth quintiles were associated with a higher probability of having an episode of diarrhea. Regarding the age of the child, the biomedical literature reports that after six months of age children change their feeding pattern by including solid foods in their diet to complement breastfeeding and the use of bottles, which could lead the child to be exposed to contaminated food, and a decrease in antibodies that are transferred from the mother during breastfeeding (39, 40). In addition, these children are exposed to contaminating factors because they are in a developmental process, during which children begin to crawl and explore their environment, thereby generating greater contact with contaminated surfaces and an increase in the prevalence of childhood diarrhea (41). Another predisposing factor for childhood diarrhea was being male, which agrees with previous studies. However, the reason for the difference in the prevalence of diarrhea between boys and girls remains uncertain (42, 43). This uncertainty has led to the creation of hypotheses related to environmental, cultural (boys have greater contact with surfaces outside the home, generating greater exposure to pathogens), and biological (greater susceptibility) factors that could lead to a higher prevalence of diarrhea in boys (42, 43).

In relation to the wealth index, previous studies have described that household wealth is related to access to sanitation and drinking water services, with poorer or poorest households more likely having unimproved sanitation and drinking water supplies, leading children to greater susceptibility to infections, such as childhood diarrhea (38, 44). Specifically, our inequality decomposition analysis corroborates these findings, identifying that the prevalence of childhood diarrhea was concentrated in those with poorer wealth index, which is consistent with studies conducted in LMIC (45). In addition, this finding could be attributed to the poorest people not using soap for handwashing (46), the lack of strategies to reduce contamination of the water they consume, or lack of access to health care services for adequate child monitoring at an early age (47), all of which could contribute to an increase in the prevalence of diarrhea.

On the other hand, our analysis of inequalities found that the major contributors to this inequality gap between the poor and rich were being 0–23 months old, belonging to the poorest and poor wealth index, and residing in the Highlands and Jungle regions. These contributors are similar to those reported in a study conducted in LMIC, in which low-income communities were reported to have 170% more cases of diarrhea compared to high-income households, and households located in rural areas (which have similar characteristics to the Highlands and Jungle regions) have 17% more cases of diarrhea compared to urban areas (45). However, this study also reported that the age of the child had the lowest contribution, likely because the age group considered children aged 12–59 months (45), while our study included children aged 0–59 months. Previous studies have described that children aged 6–11 months have a 4-fold greater risk of developing an episode of diarrhea compared to older children (48).

As for residing in the Jungle and Highlands of Peru, the National Center for Epidemiology, Prevention and Disease Control (CDC Peru) has reported that the highest incidence of diarrhea cases in children under 5 years of age in 2020 was observed in the departments of Loreto (2208.05 cases), Ucayali (2083.48 cases), Pasco (1685.89 cases), and Amazonas (1629.55 cases) (49), which is corroborated by the results of our spatial analysis, which showed that the highest numbers of diarrhea were mainly concentrated in the departments of Loreto, Ucayali, Amazonas, and Pasco. Furthermore, our study showed a higher occurrence of childhood diarrhea in these regions of the Jungle (Loreto, Ucayali, and Amazonas), confirming the information provided by the CDC of Peru (49). In addition, a previous study conducted in the Peruvian Jungle indicated that people residing in this region were exposed to various constraints or factors that increased the probability of presenting an episode of diarrhea. These factors are related to the consumption of contaminated food, lack of handwashing, consumption of unboiled water, and living in overcrowded conditions, which generate a higher risk of childhood diarrhea in this region (50). Additionally, the Jungle and Highlands regions lack a potable water supply and sanitation services, which could result in people requiring inadequate water storage for activities such as handwashing, and consequently leading to contaminated water-related diseases, such as diarrhea (51).

On the other hand, our study identified that children of mothers belonging to the 35–49 year age group, residing in a rural area, and having an improved drinking water supply were protective factors against presenting an episode of diarrhea. Regarding the age of the mother, our findings agree with those reported in studies conducted in the sub-Saharan African region (29), where children of mothers aged 35 years or older were less likely to present diarrhea. This finding could be attributed to older women’s greater experience of care, understanding and knowledge of diarrheal diseases compared to their counterparts (29). When it comes to drinking water supply, it was found that having an improved supply decreased the likelihood of childhood diarrhea, which is similar to the results of studies performed in Ethiopia (41). This finding could be due to the fact that access to adequate water supplies improves personal hygiene, mainly proper handwashing (52). Furthermore, in Peru, one study reported that water chlorination (better quality) decreased the probability of the appearance of enteropathogenic bacteria in water supplies, inducing a lower risk of presenting diarrhea in the child population (35). On the other hand, our study evidenced that residing in a rural area decreased the probability of diarrhea, which differs from studies conducted in LMIC such as Ethiopia (30). This difference could be attributed to the various programs implemented by the Peruvian government, such as the “Programa Nacional de Saneamiento Rural” (53), the “Directiva Sanitaria para la Promocionar el lavado de manos social como práctica saludable en el Perú” (54), and the “Plan Nacional de Recursos Hídricos” (55), which may have had a positive impact on population health, decreasing the risk of childhood diarrhea in rural and remote areas.

Our findings have implications for public health. Regarding public health policies, the strategies implemented by the Peruvian government should redouble efforts to allow access to sanitation services and quality drinking water in all households (regardless of socioeconomic level) and promote educational strategies on handwashing techniques in young women. These factors are included within the WASH (Water, Sanitation, and Hygiene) strategy, and there is evidence that compliance with this strategy in LMIC decreases the incidence of cases of childhood diarrhea (56). In addition, regional governments, especially in the Jungle departments of Peru, should focus efforts on reducing the lack of access to quality water in these regions, as well as educational strategies to improve water storage and food washing techniques.

The present study used a nationally representative database, which allows our results to be extrapolated to the entire Peruvian population. Furthermore, it is the first study that determines the associated factors, socioeconomic inequalities and spatial analysis of the entire Peruvian child population with updated data. Specifically, the socioeconomic inequalities in childhood diarrhea, which could be a useful tool for decision makers when focusing strategies to reduce the incidence of diarrhea in Peruvian children under 5 years of age, especially in poor people. On the other hand, our study is not without limitations. First, there is an absence of causality between the independent variables and the presence of childhood diarrhea due to the cross-sectional design of the study. Second, there could be a recall bias of the interviewees because the information obtained was based on events that occurred at specific times. Finally, some variables that could be useful for understanding the behavior of childhood diarrhea in Peru could not be determined due to the lack of availability. These variables are related to the severity of diarrhea, health status of the child, presence of parasites in feces or distance between the home and the water supply. Regarding handwashing, it should be considered that the ENDES only allows estimating this variable based on the use of soap or water; however, the medical literature states that this technique does not delimit the practice of handwashing at very important times (48), and thus, its inclusion would not be useful in the present study.

In conclusion, it was found that 10% of Peruvian children under 5 years of age had had an episode of diarrhea in 2021. In addition, we identified some sociodemographic determinants of children and household characteristics that increase the likelihood of childhood diarrhea. Childhood diarrhea had a pro-poor orientation, in which the greatest contributors were associated with age and household characteristics, while a significant positive spatial autocorrelation was found in the spatial analysis and the clusters with the highest frequency of childhood diarrhea were located in the Highlands and Jungle regions. In this sense, the scope of government strategies on access to water, sanitation, and handwashing services should include the regions with the highest prevalence of this disease (mainly the Jungle region) and all households regardless of socioeconomic status.

## Data availability statement

The original contributions presented in the study are included in the article/Supplementary material, further inquiries can be directed to the corresponding author.

## Author contributions

AH-V: conceptualization, data curation, formal analysis, investigation, project administration, and supervision. AH-V and ET: methodology and software. AH-V, ET, and RV-F: validation, visualization, writing—original draft, and writing—review and editing. All authors contributed to the article and approved the submitted version.

## Conflict of interest

The authors declare that the research was conducted in the absence of any commercial or financial relationships that could be construed as a potential conflict of interest.

## Publisher’s note

All claims expressed in this article are solely those of the authors and do not necessarily represent those of their affiliated organizations, or those of the publisher, the editors and the reviewers. Any product that may be evaluated in this article, or claim that may be made by its manufacturer, is not guaranteed or endorsed by the publisher.
