# Supplementary material for: Determinants, inequalities, and spatial patterns of diarrhea in the Peruvian under-five population: findings from nationally representative survey data
Source: Front Public Health. 2023 Jun 27;11:1170670. doi: 10.3389/fpubh.2023.1170670 (PMC10333518; doi:10.3389/fpubh.2023.1170670)
Supplement: Supplementary file 1 [file Table_1.DOCX]

Supplementary Material

Determinants, inequalities and spatial patterns of diarrhea in the Peruvian under-five population: Findings from nationally representative survey data

**Akram Hernández-Vásquez**^*^**, Rodrigo Vargas-Fernández, Efrain Y. Turpo Cayo**

*** Correspondence:** Akram Hernández-Vásquez: ahernandez@usil.edu.pe

# Supplementary Figures and Tables

## Supplementary Figures


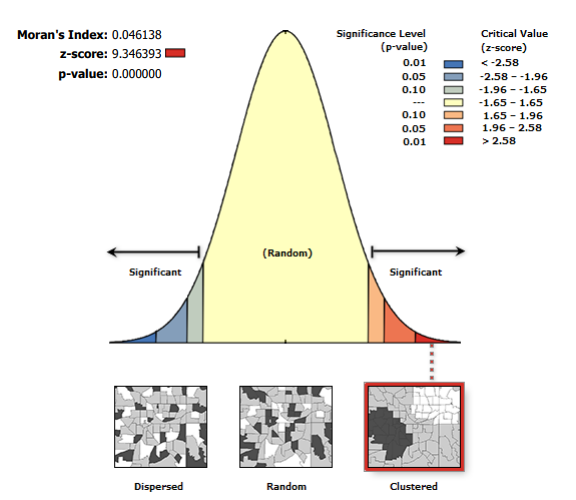


**Supplementary Figure 1.** Spatial Autocorrelation Report. Given the z-score of 9.34639265371, there is a less than 1% likelihood that this clustered pattern could be the result of random chance.


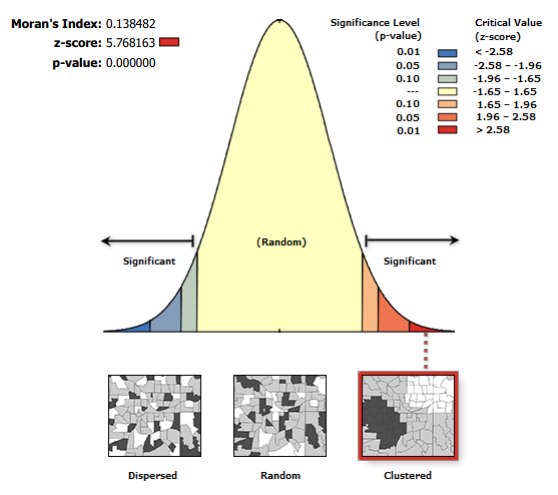


**Supplementary Figure 2.** Spatial Autocorrelation Report at district level. Given the z-score of 5.76816344998, there is a less than 1% likelihood that this clustered pattern could be the result of random chance.
